# Real-world clinical decision of andexanet alfa administration for intracranial hemorrhage during anticoagulant therapy using factor Xa inhibitor

**DOI:** 10.2478/jccm-2025-0046

**Published:** 2026-01-30

**Authors:** Shigeo Yamashiro, Keisuke Harada, Shunsuke Izumi, Yusuke Morikawa, Tomoko Ikemoto, Koki Kameno, Tomoaki Goto, Yuki Ohmori, Masatomo Kaji, Akitake Mukasa

**Affiliations:** Division of Neurosurgery, Department of Cerebrovascular Medicine and Surgery, Saiseikai Kumamoto Hospital, Kumamoto, Japan; Department of Neurosurgery, Faculty of Life Sciences, Kumamoto University, Kumamoto Japan

**Keywords:** andexanet alfa, factor Xa inhibitor, intracranial hemorrhage

## Abstract

**Introduction:**

Andexanet alfa shows excellent hemostatic efficacy in treating intracranial hemorrhage (ICH) during Xa inhibitor therapy. However, its optimal use remains uncertain.

**Aim of the study:**

This study aims to evaluate our clinical experience in managing Xa inhibitor-related ICH to clarify its appropriate application.

**Material and methods:**

This study was conducted as an observational, non-interventional study. We observed 63 cases of ICH in patients receiving anticoagulation therapy with apixaban, rivaroxaban, or edoxaban. After excluding 14 patients due to fatal outcomes or complete hemostasis, 49 patients were eligible for andexanet alfa administration.

**Results:**

The mean age and hematoma volume was 78 years and the 35ml, respectively. Based on patient characteristics and severity, andexanet alfa was administered to 23 patients, while 26 patients received usual care. Hemorrhage enlargement was absent in 22 cases (92.8%) in the andexanet group and in 22 cases (84.6%) in the usual care group. Hemorrhage expansion occurred in three cases from the usual care group, one patient undergoing emergency surgery and another died from uncontrollable intraoperative bleeding. Two patients (8.7%) in the andexanet group experienced thromboembolic events as adverse reactions. At 3 months, the modified Rankin Scale (mRS) was 3 or lower in 39% of the andexanet group and 50% of the standard care group.

**Conclusions:**

Although patient selection bias make it difficult to draw definitive conclusions, we recommend considering andexanet alfa administration for cases within several hours of the last Xa inhibitor dose to prevent neurological deterioration. Emergency surgical cases should also be eligible for andexanet alfa to ensure intraoperative safety. Further research is required to determine clinically appropriate indications for its use.

## Introduction

The number of patients, particularly the elderly, taking anticoagulants for secondary prevention of thromboembolic diseases continues to rise. While anticoagulants reduce the risk of thromboembolic events, they also increase the likelihood of hemorrhagic complications, with ICH often carrying a poor prognosis [1]. Clinicians frequently use four-factor prothrombin complex concentrates (4F-PCC) to manage bleeding associated with warfarin or direct oral anticoagulants (DOAC) [2,3]. Idarucizumab is available for the reversal of dabigatran-induced bleeding in emergency situations [3,4]. However, as most anticoagulants currently used are factor Xa inhibitors, such as apixaban, rivaroxaban, and edoxaban, there is a significant demand for a reversal agent targeting these drugs [5]. Since 2022, Japanese clinicians have had access to andexanet alfa, a reversal agent for factor Xa inhibitors[6,7]. In this study, we evaluated the clinical use of andexanet alfa and patient outcomes following acute reversal treatment for ICH in individuals taking factor Xa inhibitors at our institution. We also discuss the efficacy and indications for this new reversal treatment based on our clinical experience.

## Methods

This study is an observational, non-interventional study. We reviewed a consecutive series of 63 cases of spontaneous and traumatic ICH under the influence of apixaban, rivaroxaban, or edoxaban over a two-year period from 2022 to 2024. Enoxaparin is not available in Japan. We excluded cases of aneurysmal subarachnoid hemorrhage and chronic subdural hematoma due to their differing management strategies. Based on the results of phase III clinical trials, andexanet alfa was typically administered within 18 hours of the patient’s last DOAC dose [7]. However, the decision regarding andexanet alfa administration was made based on a comprehensive assessment of each patient’s characteristics, including age, chronic conditions, hematoma severity (volume and expansion), and life circumstances such as a living will, family wishes, and socio-economic considerations.

The method and dosage of administration were tailored to the specific factor Xa inhibitor, its dosage, and the time elapsed since the last dose. Two protocols were used: protocol A (400 mg intravenous bolus followed by a 480 mg infusion) or protocol B (800 mg bolus followed by a 960 mg infusion). Surgical indications for ICH followed the Japanese Stroke Guidelines, regardless of whether andexanet alfa was administered [3].

Data collected included patient age, gender, type of Xa inhibitor, concomitant antithrombotic agents, time from last DOAC dose to CT, as well as the type, location, and volume of the ICH. Hematoma expansion was defined as an increase of more than 20% in hematoma volume on CT scans taken 12 to 24 hours after andexanet alfa administration. Adverse events occurring within 30 days of administration, if potentially drug-related, were also recorded. Reasons for non-administration were documented when andexanet alfa was not given. The Institutional Review Board of our hospital approved this study (approval number 1055).

## Results

**Fig. 1.**
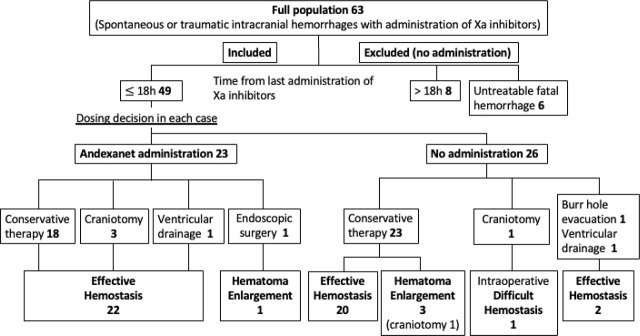
Clinical course of the study cases

The study included 63 patients with a mean age of 78.3 years, comprising 39 males and 24 females. Of these, 40 cases involved spontaneous hemorrhages and 23 involved traumatic hemorrhages. The mean hematoma volume was 34.9 ml. After excluding cases where more than 18 hours had passed since the last DOAC dose and those involving untreatable fatal bleeding, 49 cases were considered for andexanet alfa administration. Based on clinical decisions made in the emergency setting, andexanet alfa was administered in 23 cases (andexanet group), while 26 cases received usual care. The mean hematoma volume was 45.5 ml in the andexanet group while 15.4 ml in the usual care group (Table 1). Figure 1 outlines the clinical course of the study cases.

**Table 1.** Clinical characterisctics of patients

		**Andexanet**	**Usual care**
Number of patients		23	26
Mean age		78.4	76.6
Gender (M/F)		8/15	14/12

Past history			
	Hypertention	23	18
	Atrial fibrillation	18	3
	Cerebrovascular disorders	10	8

Location of spontaneous hemorrhage			
	Basal ganglia	8	12
	Cerebellum	3	1
	Pons	2	0
	Subcortex	1	1

Diangnosis of traumatic hemorrhage			
	Contusional hematoma	5	2
	Acute subdural hematoma	4	7
	traumatic SAH	0	3

Type of DOAC			
	Apixaban	5	9
	Rivaroxaban	8	4
	Edoxaban	10	13

Mean time from last dose to CT (hours)		9.7	9.6
	1 to 10 hours	14	15
	11 to 18 hours	9	11

Hematoma volume (mL)		45.5	15.4
	< 30 mL	10	21
	30–80 mL	8	30
	> 80 mL	5	1

Hematoma expansion or event of difficult hemostasis		1	4
Thromboenbolic events		2	-
Outcome (mRS)	0 to 3	9	13
	4 and 5	11	10
	6	3	3

**Fig 2.**
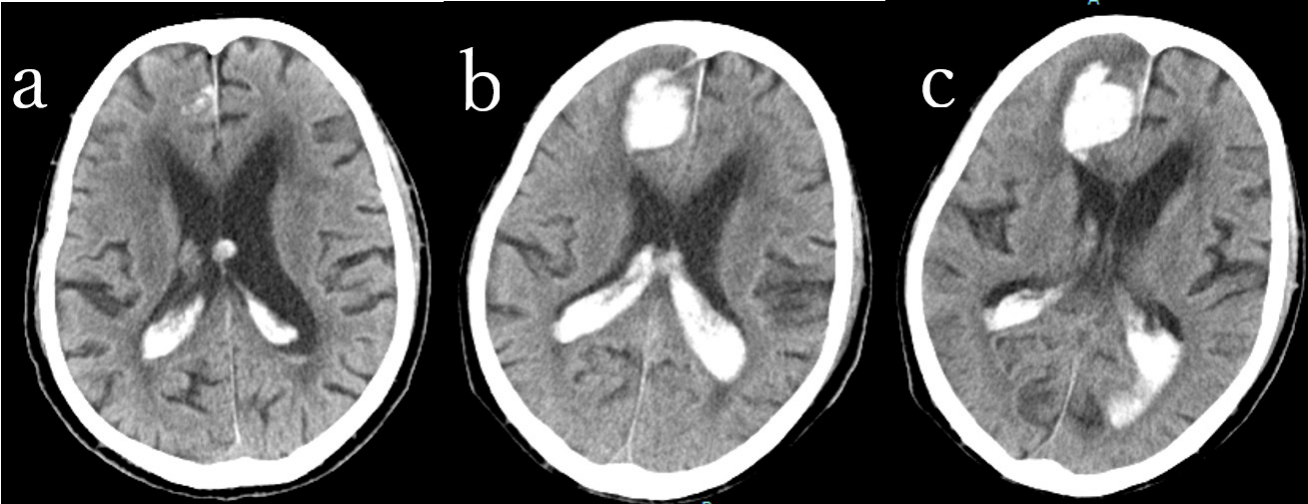
88 year-old male with rivaroxaban intake. Traumatic intracerebral and intraventricular hemorrhages. Andexanet alfa was administered after hematoma expansion was observed at day 1. a: at ambulance, b: Day1 Right frontal intracerebral hematoma was enlarged., c: Day2, No hematoma expansion 24h after andexanet administration

In the andexanet group, 23 patients (95.7%) showed no hematoma expansion. Similarly, 22 patients (84.6%) in the usual care group, including two cases treated with burr-hole surgery, also exhibited no expansion. Although the hematoma was small at the time of emergency admission and administration of andexanet alfa was deferred, it was later administered after hematoma expansion was observed the following day (Figure 2). Hematoma expansion occurred in only one patient in the andexanet group who underwent endoscopic hematoma removal for a putaminal hemorrhage.. In contrast, hematoma expansion occurred in three cases in the usual care group: cerebellar hemorrhage (7 ml), thalamic hemorrhage (20 ml), and contusional hematoma (1 ml), occurring 7, 2, and 7 hours after the last DOAC intake, respectively. The cerebellar hemorrhage case required surgical evacuation for life-saving measures, while the thalamic hemorrhage case was fatal. Additionally, one patient undergoing emergency surgery for an acute subdural hematoma, 10 hours after the last DOAC dose, died due to uncontrollable intraoperative bleeding.

Regarding outcomes at 3 months post-onset, 9 patients (39%) in the andexanet group had a modified Rankin Scale (mRS) score of 3 or less, 11 patients (48%) had an mRS of 4 or 5, and 3 patients (13%) had an mRS of 6. In the usual care group, 13 patients (50%) had an mRS of 3 or less, 10 patients (38%) had an mRS of 4 or 5, and 3 patients (12%) had an mRS of 6 (Table 1).

**Fig. 3.**
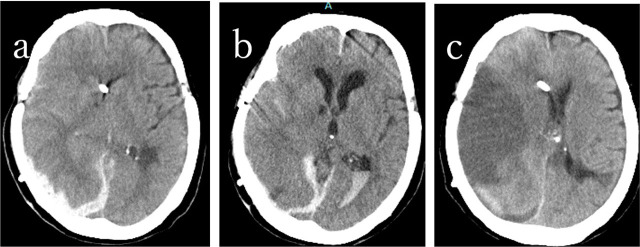
82 year-old female with edoxaban intake. Right acute subdural hematoma. Andexanet administration. a: at ambulance, b: 14h after andexanet administration, c: 14 days after the ambulance.

Adverse events following andexanet alfa administration included one case of hypertensive crisis and two cases of thromboembolic events (Table 1). Ischemic events included cerebral infarction on day 14 and arterial occlusion of the lower limb on day 8 post-administration (Figure 3). In the case of hypertensive crisis, systolic blood pressure exceeded 250 mmHg during the andexanet alfa infusion, leading to discontinuation of the drug due to suspected adverse reactions. The hematoma was subsequently evacuated endoscopically, but postoperative re-bleeding occurred.

In the usual care group, reasons for not using andexanet alfa included small hematoma volume (9 cases), anticipated hemostasis based on the clinical time course (12 cases), hemostasis confirmed by follow-up CT (1 case), ongoing treatment for cerebral infarction (1 case), advanced age with poor prognosis (1 case), and unknown reasons (2 cases). The three cases of hematoma expansion were among the 9 cases where andexanet alfa was deemed unnecessary due to the small hematoma volume.

In addition to the use of factor Xa inhibitors, several other risk factors for intracranial hemorrhage were identified among the study population. Hypertension was present in 41 patients (65%), and 18 patients (29%) had a history of prior cerebrovascular events. Traumatic hemorrhages were associated with falls, particularly in elderly patients with impaired mobility or cognitive decline. Age-related cerebral atrophy and concomitant antiplatelet therapy were also considered contributing factors in several cases. These findings suggest that while DOACs play a significant role in hemorrhagic risk, multifactorial elements must be considered in clinical decision-making.

## Discussion

Andexanet alfa acts as a decoy protein for factor Xa, rapidly restoring coagulation by binding and inactivating Xa inhibitors in the bloodstream [6,7]. Phase III trials have demonstrated its high efficacy in achieving hemostasis, leading to better control of hematoma expansion [8,9,10]. However, its real-world application remains challenging due to several factors, such as the patient’s prognosis, risk of thromboembolic complications, and the clinician’s judgment that hemostasis may have already been achieved based on hematoma volume or the time elapsed since the last Xa inhibitor dose. From our clinical perspective, we recommend administering andexanet alfa within a few hours—up to 7 hours—after the last dose, even in cases with small hematomas, to optimize patient outcomes. In cases requiring emergency hematoma evacuation, preoperative reversal with andexanet alfa may help minimize both intraoperative and postoperative bleeding, regardless of the time since the last dose.In the andexanet group, 22 out of 23 cases (95.7%) showed no hematoma expansion, demonstrating high hemostatic effectiveness. Only one case of rebleeding, associated with incomplete dosing and surgical intervention, suggests that the full recommended dose is necessary for effective hemostasis. About 80% of the usual care group also showed no hematoma expansion, indicating that spontaneous hemostasis may occur even within 18 hours of the last dose. However, three cases of hematoma expansion and one case of uncontrollable intraoperative bleeding highlight that clinicians’ judgment alone may not always be reliable, even when the time since the last dose is shorter. In surgical cases, andexanet alfa should be considered regardless of the time since the last Xa inhibitor dose, as surgical intervention may trigger re-bleeding in areas where hemostasis has already occurred.

Adverse events following andexanet alfa administration included two cases (14%) of thromboembolic events; one patient did not resume anticoagulation therapy, while the other resumed on day 7. The literature suggests that thromboembolic events are more likely when anticoagulant therapy is delayed or not resumed, underscoring the importance of restarting anticoagulation once hemostasis is confirmed[8]. Additionally, one patient experienced transient hypertension during infusion. Hypertension was not reported as a side effect in clinical trials, and its relationship with andexanet alfa remains unclear [8,9].

Andexanet alfa demonstrated excellent hemostatic efficacy in approximately 80% of bleeding cases within 12 hours of administration, regardless of the specific Xa inhibitor used. Sub-analyses focusing on ICH showed 79% efficacy for non-traumatic bleeding and 83% for traumatic bleeding. However, nearly 10% of patients experienced thromboembolic events, and 15% had fatal outcomes[8,9,10,11]. A comparative analysis with four-factor prothrombin complex concentrate (4F-PCC) showed that andexanet achieved a 17% higher hemostatic effect and a 12% lower mortality rate, indicating a preference for andexanet alfa over 4F-PCC [12,13].

In addition to the pharmacological reversal of anticoagulation, it is essential to consider the broader clinical context in which intracranial hemorrhage occurs. Our findings suggest that factors such as hypertension [14, 15], smoking [16], high alcohol intake [17], lower blood cholesterol [18] other than antithrombotic therapy significantly contribute to bleeding risk, especially in elderly patients [19]. These elements should be integrated into risk stratification models to guide the use of reversal agents more effectively.

Moreover, the decision to administer andexanet alfa should not be based solely on hematoma volume or time since the last DOAC dose. As demonstrated in our study, hematoma expansion and intraoperative bleeding occurred even in cases deemed low-risk by clinical judgment. This underscores the need for standardized protocols that incorporate both radiological and clinical parameters to support decision-making.

While our study focused on short-term outcomes, the long-term impact of andexanet alfa on functional recovery, quality of life, and recurrence risk remains unclear. Although andexanet alfa appears to provide reliable hemostatic efficacy, spontaneous hemostasis may occur in some cases, raising questions about whether it should be administered to all patients with factor Xa inhibitor-associated intracranial hemorrhage (ICH). Furthermore, due to selection bias between the andexanet and usual care groups, direct comparison of outcomes is not feasible, limiting the strength of our conclusions. Careful assessment of hematoma expansion risk and follow-up imaging is critical when andexanet alfa is not used. Clinical decisions should also consider individual prognosis and social factors. Prospective multicenter studies with standardized follow-up protocols are needed to address these gaps and clarify optimal case selection. Comparative effectiveness research involving 4F-PCC, idarucizumab (for dabigatran), and emerging agents may help refine treatment algorithms for anticoagulant-associated ICH.
